# *FvbHLH1* Regulates the Accumulation of Phenolic Compounds in the Yellow Cap of *Flammulina velutipes*

**DOI:** 10.3390/jof9111063

**Published:** 2023-10-30

**Authors:** Jiangyi Zeng, Dingding Shi, Ying Chen, Xuemei Bao, Yuan Zong

**Affiliations:** 1 Qinghai Province Key Laboratory of Crop Molecular Breeding, Northwest Institute of Plateau Biology, Xining 810008, China; zengjiangyi@scbg.ac.cn; 2 South China Botanical Garden, Guangzhou 510650, China; sdingding17@163.com; 3 College of Education, Qinghai Normal University, Xining 810008, China; chenying@hkbu.edu.hk; 4 School of Chinese Medicine, Hong Kong Baptist University, Hong Kong 999077, China

**Keywords:** *Flammulina velutipes*, ferulic acid, transcriptome, metabolome, *FvbHLH1*

## Abstract

*Flammulina velutipes* is a renowned edible and medicinal fungus. Commercially cultivated *F. velutipes* occurs in two distinct phenotypes: white and yellow. However, the underlying mechanism contributing to the yellow phenotype and high nutritional value remain uncertain. We reconfirmed that the browning process in *F. velutipes* is attributable to melanin accumulation, although the initial yellow cap seemed unrelated to melanin. A transcriptomic and metabolomic joint analysis revealed that 477 chemical compounds categorized into 11 classes, among which 191 exhibited significantly different levels of accumulation between different phenotypes. Specifically, 12 compounds were unique to the yellow *F. velutipes*, including ferulic acid, and 3-Aminosalicylic acid. Free fatty acids and xanthine were identified as the primary compounds correlating with the yellow and oily cap. A total of 44,087 genes were identified, which were more homologous to *Pleurotus ostreatus* PC15. Structural genes such as *PAL* (phenylalanine ammonialyase), *C4H* (cinnamate 4-hydroxylase), *C3H* (Coumarin-3-hydroxylase), *AoMT* (caffeoyl coenzyme A-O-methyltransferase), and *4CL* (4-coumarate: CoA ligase) were up-regulated, thereby activating the lignin biosynthesis and metabolism pathway. Additionally, *FvbHLH1* can lead to the consumption of a huge amount of phenylalanine while generating flavonoids and organic acid compounds. Meanwhile, ferulic acid biosynthesis was activated. Therefore, this study clarifies the chemical and molecular bases for the yellow phenotype and nutritional value of *F. velutipes*.

## 1. Introduction

*Flammulina velutipes*, a member of the Basidiomycotina and Agaric families, exhibits morphological similarities in its stipe shape and coloration to *Hemerocallis citrina* Baroni. The mutant variant, *F. velutipes* (white), features a white stipe and cap. The pigmentation is largely insensitive to light exposure, an attribute advantageous for commercial sales [1]. Like other edible fungi, *F. velutipes* is nutritionally robust, characterized by a high protein-to-fat ratio and containing an array of essential amino acids for humans [2,3]. Moreover, the polysaccharides of *F. velutipes* serve as immune enhancers, stimulating somatic cells, activating lymphocytes and phagocytes, augmenting antibody production, inducing interferon synthesis, and potentiating tumor growth inhibition via systemic immunomodulatory actions [4,5]. Given its short cultivation cycle, broad consumer base, and competitive pricing, *F. velutipes* and its associated industries significantly impact rural economic development and agricultural income in various Asian regions. The yellow variant of *F. velutipes* exhibits attributes such as rapid growth, robust disease resistance, wide thermal tolerance, heightened umami flavor, and superior palatability compared to its white counterpart [6,7]. To summarize, the study of yellow *F. velutipes* is important because it holds potential for health benefits and applications in the food industry. While white *F. velutipes* is preferred in the market due to its resistance to browning and convenience for transportation and preservation, yellow *F. velutipes* is often neglected and lacks nutritional value. By conducting research on the yellow *F. velutipes*, including analyzing its yellow phenotype and molecular mechanisms, the aim is to enhance our understanding of this fungal variety, and discover its hidden advantages for human health and food technology.

In recent years, high-throughput sequencing technology has established a solid foundation for the study of *F. velutipes*. A comparative transcriptomic analysis between the FrI and FrII of different *F. velutipes,* reported 78 transcripts linked to terpene biosynthesis within the steroid biosynthetic pathway [8]. The comprehensive genome sequencing of *F. velutipes* monocytic strain L11 has identified 29 high-mobility group (HMG) genes, including a specific HMG-box transcription factor, associated with the formation of *F. velutipes* primordium [9]. To date, the research on yellow *F. velutipes* has predominantly centered on cross-breeding; there is a paucity of studies delving into its coloration and molecular mechanisms. Unlike the white variety, the yellow type secretes a yellow sticky substance on its cap. The amplification of the 16S rDNA from a yellow colony utilizing universal primers 27f and 1492r revealed a 99% sequence homology with a *C. neteri* strain [10,11]. UV-VIS and infrared spectroscopic analyses suggest that tyrosinase genes *CL520contig1*, *CL1942contig2*, *CL1942contig3*, and *CL1942contig4* in the melanin synthesis pathway were upregulated [6]. Infrared chromatograms indicated three distinct features compared to tyrosine-based melanin synthesis: a stronger peak at 2920 cm^−1^ and two adjacent minor peaks. The activity of total phenol and polyphenol oxidase in browning tissues increased, while superoxide dismutase (SOD) activity decreased. The primary pigment contributing to melanin in yellow *F. velutipes* was identified as 5,6-dihydroxyindole-2-carboxylic acid derivatives.

Phenolic acids are ubiquitously distributed in nature and are particularly abundant in traditional Chinese medicinal plants such as *Lonicera japonica*, *Angelica sinensis*, and *Ligusticum chuanxiong* [12]. In plant tissues, phenolic acids predominantly occur as conjugates with sugars, lipids, and other organic acids [13]. Phenolic acids contribute to antioxidative [14], antibacterial [15], and anti-inflammatory [16] effects. These compounds are biosynthesized through the phenylalanine and tyrosine pathway [17]. Meanwhile, phenylalanine is a substrate for the biosynthesis of other phytochemicals, such as anthocyanins, flavonoids, and lignin. Key structural genes, including *PAL*, *C4H*, *4CL* and tyrosine acyltransferase are instrumental in the biosynthesis of phenolic acids [18,19]. Notably, p-coumaric acid, ferulic acid, and sinapyl acid are vital components of the plant cell wall, primarily linked through ester bonds. *PAL* is the initial enzyme in the phenylalanine metabolic pathway, which plays a decisive role in the downstream accumulation of phenolic acids [20]. In model plants, such as *Arabidopsis* [21], tobacco [22], and tomato [23], specific MYB and bHLH transcription factors regulate the accumulation of various phenolic compounds synthesized through the phenylalanine pathway. However, due to the limited solubility of melanin, the present research primarily concentrates on the analysis of precursor phenolic compounds, which act either as substrates or antioxidants in the plant browning process.

Although substantial transcriptomic and genomic investigations have been conducted on *F. velutipes*, most research has been centered on the regulatory mechanisms underlying its effective nutrients [24] or multifunctional growth factors [25]. A comprehensive examination is needed to elucidate the key compounds responsible for the yellow cap phenotype in *F. velutipes*, along with their biosynthetic and metabolic pathways, which are still unclear. This study used a transcriptomic and metabolomic approach to identify the critical nutrients and differentially expressed structural genes in yellow *F. velutipes*, simultaneously identifying the key candidate genes that regulate the synthesis of the key compounds of yellow *F. velutipe.*

## 2. Materials and Methods

### 2.1. Plant Materials

Yellow (Main Cultivation: F8) and white (Main Cultivation: Jinbai No.1) phenotype of *Flammulina velutipes* (Agaricel, Tricholomataceae, *Flammulina*) were obtained and archived at the Northwest Institute of Plateau Biology, Chinese Academy of Sciences, Xining, China. No special permission was required for sample collection. For the sequence analysis of yellow *Flammulina velutipes*, *Flammulina velutipes* KACC42780 (basidiomycetes), BioSample: SAMN01940701, BioProject: PRJNA191921, was employed as the reference genome. Similarly, *Flammulina velutipes* (basidiomycetes), BioSample: SAMN14149291, BioProject: PRJNA603211 served as the reference genome for white *Flammulina velutipes*. *FvbHLH1* sequence was uploaded to NCBI (National Center for Biotechnology Information, Bethesda, MD, USA), and the GeneBank submission number was 2756780.

### 2.2. UPLC-ESI-Q TRAP-MS/MS Analysis of Cap

*F. velutipes* were pulverized for 1 min at 30 Hz using sterile steel balls. A total of 100.00 mg of powder was extracted overnight with 1.20 mL 70% methanol solution. Subsequent to centrifugation at 12,000 rpm for 10 min, the supernatant was analyzed using UPLC-ESI-Q TRAP-MS/MS.

Analytical conditions involved an Agilent SB-C18 column (2.1 mm × 100 mm i.d., 1.8 µm, Agilent, Santa Clara, CA, USA), with a mobile phase consisting of 0.1% formic acid (A) and 0.1% formic acid in acetonitrile (B). The gradient program initiated with a linear transition from 5 to 95% B over 0 to 9 min, then did not change for 1 min, and subsequently decreased from 95 to 5% B from 10.00 to 11.10 min, which was held constant until 14 min. The flow rate was 0.35 mL/min, temperature was 40 °C, and the injection volume was 4.00 µL.

Mass spectrometry scans were executed on a triple quadrupole linear ion trap mass spectrometer (Q-TRAP) interfaced with an AB4500 UPLC/MS/MS system (AB SCIEX, Framingham, MA, USA). The system featured an ESI Turbo Ion Spray interface and was operated in both positive and negative ion modes using Analyst 1.6.3 software (SCIEX, Vaughan, ON, Canada). Ion source settings were configured with a turbo spray source at a temperature of 550 °C, ion spray voltages of 5500 V (positive ion mode), and −4500 V (negative ion mode), and the ion source gases I (GSI), II (GSII), and gas curtain (CUR) were set to 50, 60, and 25.0 psi, respectively. The collision-activated dissociation (CAD) gas setting was high. Instrument tuning and quality calibration in QQQ and LIT modes were performed using 10 and 100 µmol/L polypropylene glycol solutions, respectively. Further optimization of DP and CE for each MRM transition was conducted based on the metabolites eluted during specific periods.

### 2.3. PCA Analysis and KEGG Annotation

Principal component analysis (PCA) was conducted using the prcomp function within the R statistical environment (www.r-project.org (accessed on 12 June 2022)). Differentially regulated metabolites between samples were ascertained based on VIP values ≥ 1 and Log2 fold change (Log2FC) ≥ 1. VIP values were collected from the results of orthogonal partial least squares discriminant analysis (OPLS-DA), which included score plots and permutation plots. This analysis was executed using the MetaboAnalystR 5.0 package in R. Data transformation was performed using Log2 and subsequently mean-centered prior to the OPLS-DA. To mitigate the risk of overfitting, a permutation test comprising 200 permutations was executed.

The identified metabolites were annotated utilizing the KEGG compound database (http://www.kegg.jp/kegg/compound/ (accessed on 18 July 2022)). These annotated metabolites were subsequently mapped onto the KEGG pathway database (http://www.kegg.jp/kegg/pathway.html (accessed on 19 July 2022)). Pathways containing significantly regulated metabolites were subsequently subjected to metabolite sets enrichment analysis (MSEA). Statistical significance was assessed using hypergeometric test *p*-values.

### 2.4. Transcriptome Sequencing and Analysis

Mature caps of white and yellow *F. velutipes* were collected, flash-frozen in liquid nitrogen, and subsequently stored at −80 °C until further use. The total RNA was extracted using the RNAprep Pure Plant Kit (DP441, Tiangen biotech, Beijing, China) and the purity of the total RNA was quantified by NanoDrop™ (Termo Scientifc, Wilmington, DE, USA). First-strand cDNA was synthesized using the PrimeScript™ II 1st Strand cDNA Synthesis Kit (Code No. 6210A, TaKaRa, Osaka, Japan) for RNA-seq.

Oligo(dT) was utilized to enrich for mRNA, followed by the random fragmentation of mRNA. The cDNA library was subsequently constructed through PCR enrichment. Quality assessment of the transcriptome sequencing library was imperative to ensure the integrity of the sequencing process. Qualified cDNA samples were then sequenced using an Illumina high-throughput sequencer (X-10). High-quality sequencing data were assembled using Trinity. The process involved the construction of a K-mer library from the sequencing reads, error-filtering of K-mers, seed extension at both ends based on the highest-frequency K-mer, and clustering of resulting contigs to construct a De Bruijn graph. Ultimately, transcript sequences were obtained.

For annotation, BLAST software (https://blast.ncbi.nlm.nih.gov/Blast.cgi (accessed on 20 June 2022)) was employed to align Unigene sequences with multiple databases, including Nr, Swiss-Prot, GO, KOG, and KEGG. After amino acid sequence prediction, HMMER software (3.3.2) was utilized to compare with the Pfam database for further annotation. Sequence reads were aligned to the Unigene library using Bowtie and the expression levels were assessed using RSEM, with FPKM values serving as indicators of Unigene expression abundance [26]. Given the biological replicates in the experiment, DEGSeq was applied for differential expression analysis between sample groups [27]. False discovery rate (FDR) was employed as a crucial metric for the screening of differentially expressed genes (DEGs) to minimize false positives. An FDR of less than 0.001 and a fold change of greater than or equal to 1 were set as the identification criteria.

### 2.5. Overexpression of FvbHLH1 in Tobacco

The full-length sequence of *FvbHLH1* was amplified via polymerase chain reaction (PCR), which was conducted in the GeneAmp PCR System 9700 (Thermo-Fisher Scientific, Shanghai, China). PrimeSTAR^®^ MaxDNA Polymerase (Code No. R045A, TaKaRa, Osaka, Japan) was used, the cDNA and gDNA of *F. velutipes* were used as a template, the 25.00 µL reaction system included 12.50 µL PrimeSTAR Max Premix (2×), 0.25 µL each primer, 11.00 µL ddH_2_O, and 1.00 µL cDNA or gDNA. The reaction program was as follows: 35 cycles at 98 °C for 10 s, 55 °C for 15 s and 72 °C for 1 min. Specific primers were designed by Vector NTI10.0 (Table S1). The obtained sequence was subsequently cloned into the pEASY^®^-Blunt vector (TransGen Biotech, Beijing, China), and transformed into *Escherichia coli* DH5α cells. Positive clones were verified through sequencing by Sangon (Shanghai, China). Double digestion of the *FvbHLH1* and PC2300s plasmids was performed using the restriction enzymes *Xba*I and *BamH*I (TaKaRa, Osaka, Japan). The PC2300s plasmids were provided by Qinghai Province Key Laboratory of Crop Molecular Breeding (Xining, China). Recombinant plasmid PC2300s: *FvbHLH1* was transformed into *Agrobacterium tumefaciens GV3101*, which was cultivated in LB solid media containing Kana (100.00 mg/mL) and Rif (100.00 mg/mL) at 28 °C for 48 h. The leaf disc method was applied for subsequent tobacco transformation [28]. *A. tumefaciens* infection solutions (OD600 = 0.6–0.8) were prepared to infect tobacco leaf (0.50–1.00 cm^2^); the infection time was 6–8 min. Subsequently, these plants were transferred to MS medium with a dark treatment for 2 d. Then, tobacco calluses were transferred to 6CN media containing 6−benzylamino purine (1.00 mg/mL), cefotaxime sodium (100.00 mg/mL), and 1−naphthaleneacetic acid (1.00 mg/mL) for 7 d. Callus differentiation culture was conducted in 6CNK (100.00 mg/mL kanamycin) media for 7 d. Next, the differentiated shoots were transferred to CNK media for about 14 d until the shoots took root. The transgenic shoots grow in the incubator with long-day lighting (16 h light/8 h dark). The nutrient media needed periodic replacement.

### 2.6. qRT-PCR

Quantitative reverse transcription PCR (qRT-PCR) was conducted using the TB Green^®^ Premix Ex Taq™ (Tli RNaseH Plus) (Code No. RR420Q, TaKaRa, Osaka, Japan) on an Applied Biosystems Quant Studio system (Thermo Fisher Company, Beijing, China). The 20.00 µL reaction system included 10.00 µL TB Green Premix Ex Taq (Tli RnaseH Plus) (2×), 0.40 µL primer (10 µM), 0.40 µL ROX Reference Dye (50×), 2.00 µL cDNA and 6.80 µL ddH_2_O. The qRT-PCR reaction procedure was as follows: 1 cycle at 95 °C for 30 s, 40 cycles at 95 °C for 5 s, and 55 °C for 34 s, followed by 1 cycle at 95 °C for 15 s, 60 °C for 1 min, and 95 °C for 15 s. The specific primer sequences are shown in Table S1. The relative expression level was quantified by the 2^−∆∆CT^ method and the experiment was replicated three times to ensure biological validity. Protein–protein interactions were predicted using the PPA-Pred 2 (protein-protein affinity predictor) (http://www.iitm.ac.in/bioinfo/PPA_Pred/ (accessed on 20 April 2023)).

### 2.7. Total Melanin Extraction

0.20 g samples were extracted with 5.00 mL 1 mol/L NaOH and incubated for 30 min at 100 °C. The reaction mixture was acidified using 6 mol/L HCl, followed by the addition of n-butanol to extract pigments. The absorbance at 400 nm was determined to quantify pigment concentration.

### 2.8. Determination of Ferulic Acid

A standard solution of ferulic acid (6.50 mg; CAS#83-88-5, HPLC ≥ 99%, experimental concentration 1%, Yuanye Biology, Shanghai, China) was dissolved in 10.00 mL methanol. Fresh transgenic tobacco leaves (0.70 g) were extracted with 2.00 mL 75% methanol. Then, ultrasound was performed at 50 °C for 30 min, followed by centrifugation at 4000 r/min for 10 min. After flotation through a 0.22 µm filter, the samples were retained for HPLC analysis. Analysis was performed using an Agilent HPLC system (Agilent, Santa Clara, CA, USA) with a Kromasil C18 column (4.6 mm × 250 mm i.d., 5 µm, Kromasil, Gothenburg, Sweden). The mobile phase comprised 0.5% trifluoroacetic acid and acetonitrile, employing an isocratic elution program of 70% of 0.5% trifluoroacetic acid and 30% acetonitrile for 30 min. The flow rate was 1.00 mL/min, the column temperature was 30 °C and the injection volume was 80.00 µL, the detection wavelength was 324 nm. The standard curve equation was Y = 4E + 0.6X + 14.457, R^2^ = 1.

### 2.9. UPLC-ESI/MS Analysis

A total of 0.20 g fresh transgenic tobacco leaves were extracted with 2.00 mL 75% ethanol. The sample was subjected to ultrasound at 50 °C for 30 min and centrifuged at 4000 rpm for 10 min. The supernatant was filtered through a 0.22 µm filter for analysis. Analysis was performed with Thermo Scientific TSQ Endura LC-ESI-MS system equipped with a UPLC Hypersil Gold column (100 × 2.1 mm, 1.9 µm), the flow rate was 0.20 mL/min. The mobile phase consisted of 0.1% formic acid (A) and acetonitrile (B) in a gradient elution. Detection wavelengths were set at 268 nm and 290 nm. The mass spectrometer parameters were configured as outlined.

### 2.10. Primary Metabolomics of Transgenic Tobacco

Freeze-dried transgenic tobacco leaves were processed using a vacuum freeze-drying machine (Scientz-100F, Ningbo Xinzhi Biotechnology, Ningbo, China) and ground into a fine powder at 30 Hz for 1.5 min using a grinder (MM 400, Retsch company, Arzberg, Germany). A total of 50.00 mg of powder were extracted with 1.00 mL 70% methanol containing an internal standard. Petroleum ether (0.50 mL) was added, followed by vortexing for 5 min and centrifugation at 12,000 rpm for 10 min at 4° C. The supernatant was passed through a 0.22 µm PTFE filter prior to UPLC-MS/MS analysis. The UPLC-ESI-MS/MS system and conditions were consistent with those used for *F. velutipes*.

### 2.11. Statistical Analysis

Data are represented as the mean ± standard deviation. The experiment was performed with three biological repeats. GraphPad Prism 8.0 software (GraphPad Software, Inc., San Diego, CA, USA) was used to perform statistical analysis. Significant differences were determined based on the results of Student’s *t*-test and one-way ANOVA.

## 3. Results

### 3.1. Chemical Compound Differences in White and Yellow F. velutipes

**Figure 1:**
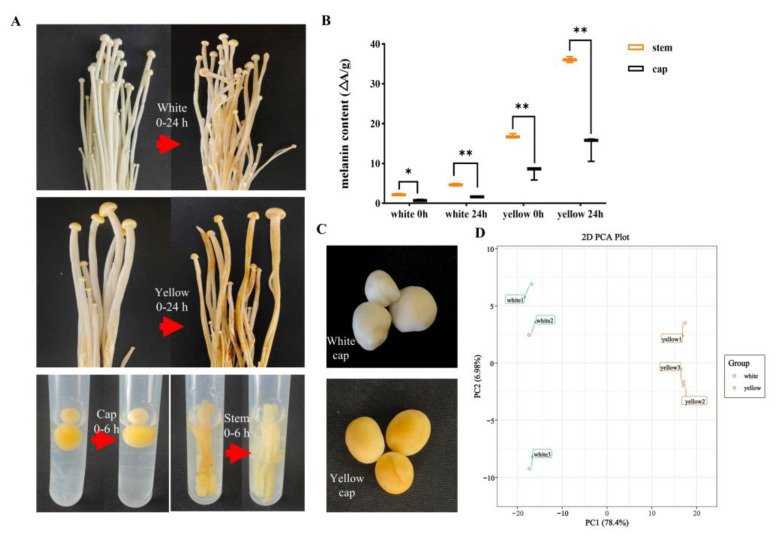
The yellow and white phenotype of *F. velutipes*. Discoloration appearance (**A**) and melanin content (**B**) of *F. velutipes* (Yellow and white) mushroom after stored at room temperature for 0, 6, and 24 h. (**C**) The white and yellow caps of *F. velutipes*. (**D**) Principal component analysis of all the samples. Values are shown as means ± SD. (Student’s *t*-test, * *p* < 0.05, ** *p* < 0.01, *n* = 3).

The cap and stem of white and yellow *F. velutipes* exhibited noticeable browning upon incubation at room temperature (25 °C) for 24 h, the yellow variant showing pronounced color changes (Figure 1A). Melanin was extracted from the cap and stem of yellow *F. velutipes*. After extracting, the stem color transitioned from brown to white, whereas the cap color remained relatively stable (Figure 1A). The initial melanin content in the yellow cap was approximately twice than that in the stems, however, after 24 h oxidation, no significant increase in melanin was observed in the caps. In contrast, the melanin content of the stems increased nearly 1.5-fold, which exhibited clear browning (Figure 1B). The MIM-EPI and RNA-seq methods were employed to elucidate the distinct metabolic profiles and key regulatory gene expression between yellow and white caps of *F. velutipes* (Figure 1C). The PCA revealed distinct metabolic profiles between yellow and white variants (Figure 1D). A total of 477 chemical compounds categorized into 11 classes were identified, with 191 compounds demonstrating significantly divergent accumulation patterns in the yellow and white lines (Figures S1 and S2), only quinones did not show a differential accumulation. The metabolite content analysis (Table S2) highlighted that the highest concentrations were of free fatty acids, followed by saccharides, organic acids, amino acids, and phenolic acids. The high concentration of these particular substances likely underpins the nutritional value of *F. velutipes*.

**Figure 2:**
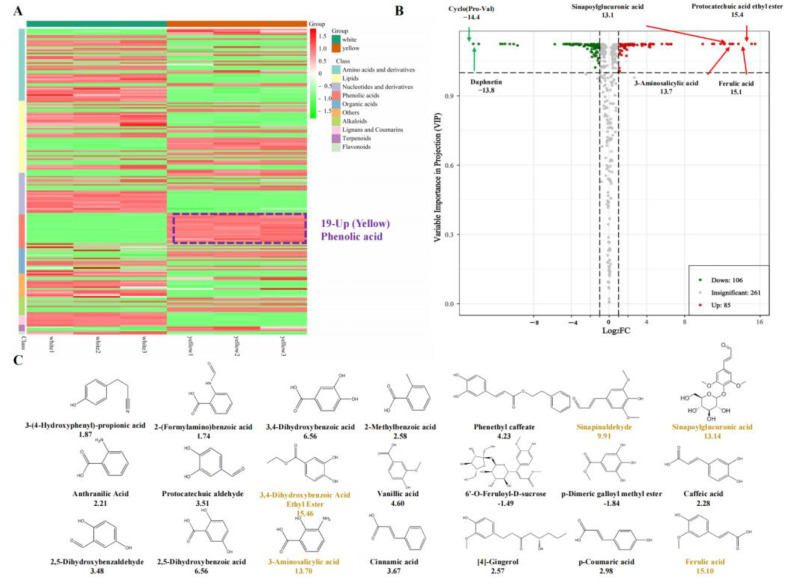
Analysis of the metabolites detected by the metabolome. (**A**) Clustered profiles of DAMs. The color scale on the right represents re-processed log10 (FPKM) using heatmap, the expression variance for each metabolite is indicated by different colors ranging from low (green) to high (red). (**B**) Volcano plots of the metabolic profiles in the white cap and yellow cap. (**C**) The 21 differentially accumulated phenolic compounds in the white cap and yellow cap.

The heat map illustrating the compound enrichment classification revealed marked disparities in phenolic acid compounds within the yellow caps (Figure 2A). A total of 85 compounds were predominantly accumulated in the yellow cap, among which phenolic acids exhibited the most significant differences (Figure 2B). The KEGG annotation of the differentially accumulated compounds indicated that the ABC transporter pathway had the greatest number of enriched compounds, succeeded by purine metabolism, phenylpropanoid biosynthesis, and arginine and proline metabolism (Figures S3 and S4). Exclusive to the yellow cap, 12 phenolic compounds were identified, including sinapinaldehyde, sinapoylglucuronic acid, 3-aminosalicylic acid, ferulic acid, and protocatechuic acid ethyl ester being the most prevalent. Out of the 35 detected phenolic acid compounds, 21 exhibited differential accumulation, 19 of which were more abundant in the yellow cap and were categorized into caffeic acid derivatives and benzoic acid derivatives. Compound ranking analysis suggested that linoleic acid, stearic acid, and elaidic acid could be the key contributors to the oily texture of *F. velutipes* caps (Figure 2C). According to the ranking analysis of differentially accumulated compounds, the yellow cap displayed elevated levels of 2,5-dihydroxybenzaldehyde, protocatechuic acid, 2, 5-dihydroxybenzoic acid, and caffeic acid, all of which were either yellow or light yellow compounds. These compounds are likely responsible for the yellow phenotype of the *F. velutipes* cap.

### 3.2. Chemical Compound Differences in White and Yellow F. velutipes

**Figure 3:**
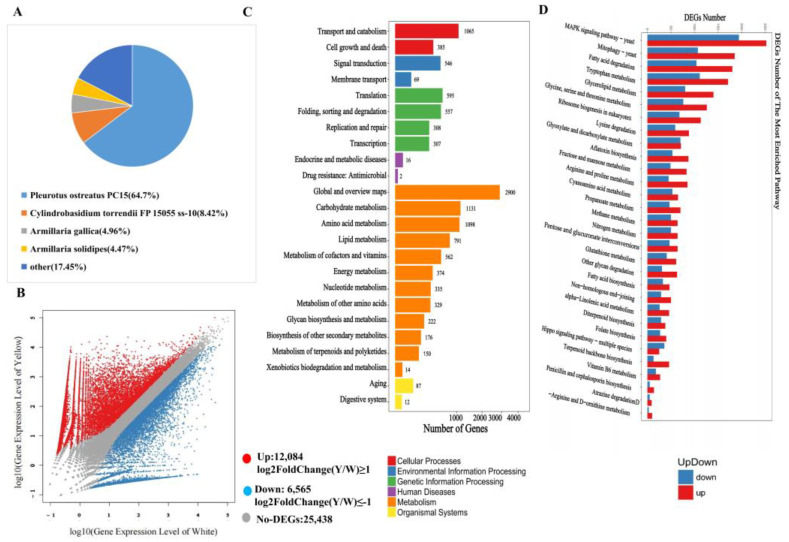
Gene expression analysis of RNA-seq samples. (**A**) Protein annotation and classification. (**B**) Volcano plot of DEGs in *F. velutipes* (Yellow caps and white casp). Each point represents a DEG. Genes with log10 FC above 1 were considered to be up-regulated genes, shown in red. Genes with 10-based log fold change below −1 were considered to be down-regulated genes, shown in blue. The genes in gray show no differential expression. (**C**) KEGG pathway of differentially expressed unigenes. (**D**) Top 30 KEGG pathways enriched in the annotated DEGs.

Three biological replicates of white and yellow *F. velutipes* were selected for sequencing, yielding a total of 242.31 million raw reads. The RNA-seq results demonstrated that the Q20 and Q30 values exceeded 98% and 95%, respectively (Table S3). The assembly process generated more than 27,000 unigenes for white and 34,000 unigenes for yellow *F. velutipes*, with a comprehensive assembly of 191,852 unigenes. These unigenes ranged in size from 297 bp to 14,979 bp (Table S4). All the assembled genes were annotated using multiple protein databases, including KEGG, NR, KOG, SwissProt, and Pfam (Table S5). The homologous comparison revealed that 64.7% proteins shared similarities with *P. ostreatus PC15* (Figure 3A). The Log2FC analysis indicated that 12,084 genes exhibited higher expression in yellow *F. velutipes*, whereas 6565 genes were more highly expressed in white *F. velutipes* (Figure 3B). The KEGG annotation results predominantly annotated the genes in three categories: transport and catabolism (1065 genes), carbohydrate metabolism (1131 genes), and amino acid metabolism (1098 genes) (Figure 3C and Table S6). Among the 30 primary metabolic pathways, the yellow cap displayed a higher number of DEGs, notably in pathways such as the MAPK signaling pathway, mitophagy, fatty acid degradation, and tryptophan metabolism. This was also evident in multiple synthetic metabolic pathways involving amino acids such as arginine, lysine, glycine, and serine (Figure 3D and Table S7).

### 3.3. Conjoint Analysis of Transcriptome and Metabolome

**Figure 4:**
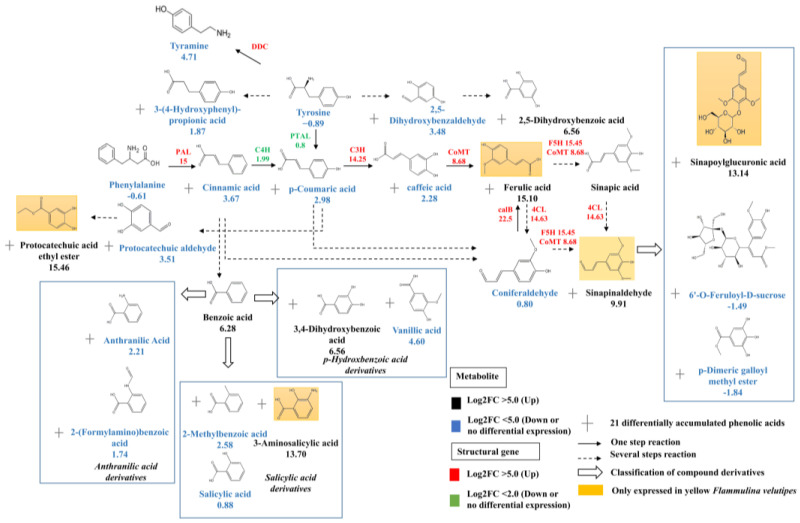
The DEGs and DAMs in biosynthesis pathway. Arrows show the metabolic stream; left or upward arrows represent the genes catalyzing the progress. The red abbreviations express the genes found in the assembly unigenes, and the number under the genes represents the relative expression level in the yellow cap versus the white cap. The numbers under the chemical compounds represent the relative contents of the compounds accumulated in the yellow cap versus the white cap.

To evaluate the implications of transcriptomic alterations on the metabolome, DEGs, and variably accumulated compounds were mapped onto KEGG pathways (Figure 4). A total of 29 pathways contained both DEGs and differentially accumulated compounds. In 26 of these pathways, the enrichment of DEGs was significant with *p*-values less than 0.01, with the exceptions of carbapenem biosynthesis, metabolic pathways, and selenocompound metabolism, of which the *p*-values were below 0.05. The enrichment scores for all compounds in these 29 pathways also exhibited *p*-values less than 0.05, notably, compounds involved in the sulfur relay system pathway were highly enriched. The KEGG annotation illustrated that the 21 types of phenolic acid compounds predominantly localized within the pathways for phenylalanine, tyrosine, and tryptophan biosynthesis. Among these, caffeic acid derivatives were primarily involved in the lignin anabolic pathway. Specifically, phenylalanine served as the substrate in a sequence of reactions that produced ferulic acid and its downstream product, sinapoylglucuronic acid both exclusively detected in the yellow cap. The conjoint analysis revealed the upregulation to varying extents of structural genes involved in lignin synthesis, including *PAL* (15), *PTAL* (0.8), *C4H* (1.99), *C3H* (1.36), *AoMT* (13.52), *calB* (10.07), *F5H* (15.45) and *4CL* (14.36). Notably, the Log2FC values for *PAL*, *AoMT*, *calB*, *F5H* and *4CL* exceeded 10. The further clustering of the differentially expressed phenolic acid compounds in yellow *F. velutipes* demonstrated that some compounds primarily initiated with phenylalanine as the substrate. Meanwhile, others were primarily synthesized from cinnamic acid, subsequently forming benzoic acid and then generating derivatives such as anthranilic acid, salicylic acid, and p-hydroxbenzoic acid. Among this categorization, 19 differentially accumulated phenolic compounds were annotated with their chemical structures and associated with KEGG pathways (Ko00350, Ko00950, and Ko00940), excluding gingerol and phenyl buffer, which remained unannotated.

### 3.4. Transcription Factors Regulate Phenolic acid Biosynthesis

**Figure 5:**
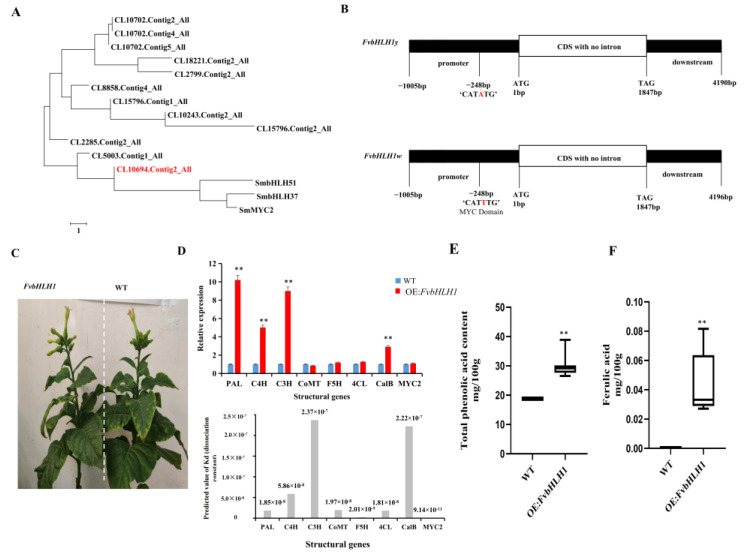
Overexpression of *FvbHLH1* increased total phenolic acid accumulation in the leaves of tobacco. (**A**) The phylogenic tree of the bHLHs from *S. miltiorrhiza* and *F. velutipes*. (**B**) Schematic diagrams of the *FvbHLH1y* and *FvbHLH1w*. (**C**) Phenotypes of wild-type and transgenic tobacco lines. (**D**) The relative transcript level of the genes relative to lignin biosynthesis and metabolism in transgenic tobacco leaves based on qRT-PCR. Values are shown as means ± SD. (Student’s *t*-test, ** *p* < 0.01, *n* = 3). (**E**) The total phenolic acid and (**F**) the content of ferulic acid.

The statistical analyses revealed there are 42 MYB and 22 bHLH transcription factors in *F. velutipes*. The key MYB and bHLH transcription factors that regulate the biosynthesis of phenolic acids in *Salvia miltiorrhiza* Bge were selected to construct a phylogenetic tree with the transcription factors identified in this study (Figure 5A). These results demonstrated that the MYB transcription factors *CL8511.Contig2_All*, *CL8511.Contig5_All*, and *CL8511.Contig6_All* exhibited homology with MYB transcription factors in other species. However, the three genes manifested low FPKM values in both white and yellow *F. velutipes*. The bHLH phylogenetic tree indicated that only *CL10694.Contig2_All* (*FvbHLH1*) showed significant relevance to the regulation of phenolic acids. *FvbHLH1* was scarcely expressed in white specimens, exhibiting an FPKM value of 11,080 and the Log2FC value was 9.24. Therefore, *FvbHLH1* was identified as a key bHLH transcription factor. The comparative analyses found no differences in the *FvbHLH1* ORF region (Figure 5), and several base differences in the promoter region between the white and yellow *F. velutipes*. However, a MYC element difference was found in the promoter region with sequences “CATTTG” in white and “CATATG” in yellow (Figure 5B). The overexpression of *FvbHLH1* in tobacco did not make significant changes to the tobacco phenotype or plant height (Figure 5C). qRT-PCR results indicated that structural genes related to lignin biosynthesis and metabolism in transgenic tobacco leaves were upregulated, notably *PAL*, *C4H*, *C3H* and *CalB*. Software prediction corroborated that *FvbHLH1* interacted to varying extents with these structural genes, with the most significant interactions observed for *C3H* and *CalB*, which were consistent with the qRT-PCR results (Figure 5D).

### 3.5. Detection of Compounds in Transgenic Tobacco

The total phenolic acids in *FvbHLH1* transgenic tobacco exhibited a significant increase, predominantly around 30.00 mg/100 g (Figure 5E). Ferulic acid, undetectable in wild-type tobacco, was present in the transgenic variants (Figure 5F). The primary metabolome sequencing of tobacco leaves revealed that the detection of 621 primary metabolites across 11 categories. Although the concentration of most primary metabolites were elevated in wild-type tobacco, the levels of 20 free fatty acids and 28 carbohydrate compounds were higher in transgenic tobacco. Based on the Log2FC value assessment, 5,8,11,14-pentadecanoamide and lysoPC 20: 0 were exclusively detected in transgenic tobacco. Furthermore, the overexpression of *FvbHLH1* predominantly promotes the accumulation of saccharides, amino acids, and coumarin acid derivatives. Conversely, phenylalanine and its derivatives were substantially down-regulated in transgenic tobacco (Figure S7).

The mass spectrometry (MS) analysis of tobacco extracts revealed multiple differentially expressed compound peaks, with the most significant ones observed at 5.88, 9.63, and 21.5 min in transgenic tobacco. The molecular weights indicated that compounds at different time points were primarily flavonoids, which were more abundant in the transgenic lines. Conversely, the concentration of one compound decreased to near-zero levels at 21.5 min in transgenic tobacco and its molecular weight was predicted to be phenylpropanoids (podophylloxin). Additionally, a compound with elevated levels compared to the wild-type was identified at 16.25 min; it might be an organic acid or derivative thereof (4-O-caffeoyl quinic acid, chlorogenic acid, 1-O-caffeoyl quinic acid).

## 4. Discussion

The yellow cap and white cap distinguish two different flavors and values of *F. velutipes*, and there is still no clear article reporting the differences. We have identified the critical compounds responsible for the phenotypic and nutritional variations between the two cap colors, as well as the key candidate genes involved in the biosynthetic pathway of phenolic pigments. Various yellow derivatives, such as 2, 5-dihydroxybenzaldehyde, ethyl protocatechuic acid, and ferulic acid, may be key compounds in the yellow cap, different from the browning of *F. velutipes.* Specifically, the gene *FvbHLH1* was uniquely expressed in the yellow cap and is posited to play a regulatory role in phenolic acid biosynthesis.

### 4.1. The Pigment Composition and Color Variation in Yellow F. velutipes

The commercial cultivation of *F. velutipes* began in Japan and China [29], but there are no definitive reports on the pigment composition of yellow *F. velutipes*. Previously, studies on other fungi suggested that melanin may affect the color change in *F. velutipes* [30]. The browning of the stipe is due to enzymatic reactions using phenolic compounds as substrates. Unique phenolic acid compounds in yellow *F. velutipes* contribute to the melanin synthesis. A 24 h experiment showed the stipe turning from light yellow to dark brown, which correlated with an increase in 3,4-dihydroxy-phenylalanine. Melanin was extracted from both the stipe and cap of yellow *F. velutipes*, resulting in the color change of the stipe from brown to white (Figure 1A,B), which suggested that other yellow compounds may accumulate specifically in the yellow cap. Riboflavin was not responsible for the yellow in the cap [31]. These results revealed that the most abundant compounds in *F. velutipes* were free fatty acids, which align with the characteristics of the yellow and greasy cap (Figure 2A,B). Additionally, several other yellow compounds were exclusive to the yellow variant of *F. velutipes*, with 2, 5-dihydroxybenzaldehyde being significantly higher in the yellow cap compared to the white variant. Linoleic acid, elaidic acid, and stearic acid likely contribute to the greasiness of the cap rather than the yellow coloration (Figure 3A). The yellow cap phenotype of *F. velutipes* is mainly attributed to α-linolenic acid, γ-linolenic acid, protocatechuic acid ethyl ester, sinapinaldehyde, ferulic acid, and 2, 5-dihydroxybenzaldehyde.

### 4.2. Saccharides and Phenolic Acids Are the Unique Nutrients of F. velutipes

This study incorporated the metabolomic sequencing of A. *auricula* and A. *bisporus* for a comparative analysis. The unique accumulations of certain nutrients were observed in *F. velutipes*, including 1,4-Dihydro-1-Methyl-4-oxo-3-pyridinecarboxamide, L-Arabitol, Ribitol, and Xylitol, which ranked among the top 12 metabolites. Notably, L-Arabitol serves as a novel low-calorie sweetener that resists metabolism in the human body and inhibits sucrase-mediated hydrolysis, consequently reducing sucrose absorption, and blood sugar levels [32]. Xylitol also contributes to cellular nutrient and energy supplies without eliciting blood sugar spikes [28]. These attributes make *F. velutipes* a particularly advantageous food option for diabetics. Furthermore, l-arabitol is an essential component of polysaccharides [33], this advantage may drive the nutritional value of the yellow *F. velutipes* in the market.

The nutritional differences between yellow and white *F. velutipes* have remained largely unexplored. Our investigation identified key differential nutrients at the chemical level. Noteworthy compounds like 2,5-dihydroxybenzaldehyde, with hepatoprotective and anti-inflammatory properties, and xanthine, useful in treating bronchiectasis and asthma, were prevalent in the yellow variant but scarce in the white variant (Figure 2C). Furthermore, the yellow variant exclusively contained 12 compounds, including five phenolic acids: protocatechuic acid ethyl ester, ferulic acid, 3-aminosalicylic acid, sinapoylgluconic acid, and sinapinaldehyde, whereas the white variants lacked L-serine. Phenolic acids have gained significant attention in recent pharmacological research, especially within the domain of traditional Chinese medicine. For instance, the polyphenolic acid in *Salvia miltiorrhiza* has been utilized to develop anti-apoplexy medications effective for cerebrovascular diseases [34]. Clinical studies have substantiated the neuroprotective effects of phenolic acids, improving the life quality of cerebral infarction patients [35]. Among these, ferulic acid stands out for its potent antioxidant properties [36]. Collectively, this study confirms that *F. velutipes* contains an array of medicinal compounds absent in other mushrooms, thereby offering substantial benefits to human health.

### 4.3. FvbHLH1 Regulates Phenolic Acid Biosynthesis and Metabolism

A range of MYB and bHLH transcription factors identified in plants were overexpressed in both tobacco and *Arabidopsis*, providing evidence that these transcription factors induce lignin accumulation and modulate the anabolism of phenolic acids in these plants. Specifically, the overexpression of *PtrMYB3* and *PtrMYB20* of *Populus trichocarpa* [37], *PtMYB4* of *Pinus taeda* L. [38], and *EgMYB2* of *Eucalyptus grandis* Hill [39] in *Arabidopsis* activated the biosynthesis of cellulose, xylan, and lignin, which also induced the transcriptional regulation of key genes such as *C4H*, *4CL* and *CAD*. In a parallel vein, the overexpression of *SmMYC2* from *Salvia miltiorrhiza* led to the increased accumulation of rosmarinic acid (RA), and salvianolic acid B (SAB), with its interaction with Jasmonate Zim (JAZ) proteins further augmenting phenolic acid levels [40]. *SmbHLH37* has been shown to bind to the promoters of the phenolic acid biosynthetic genes *SmTAT1* and *SmPAL1*, thereby negatively regulating the biosynthesis and accumulation of SAB [41]. Based on the transcriptome analysis, 42 MYB and 22 bHLH transcription factors were identified. According to the phylogenetic tree of MYB TFs, three different transcripts of *CL8511* were screened, although their FPKM values were relatively low. The most homologous gene was a key transcription factor known to regulate phenolic acid accumulation in *Salvia miltiorrhiza* compared to the bHLH transcription factor *CL10694.Contig2_All* (*FvbHLH1*) (Figure 5A). *FvbHLH1* was not expressed in the yellow cap and its MYC element was notably different. Accumulating evidence indicates that numerous regulatory elements of gene expression are located within the promoter, most of which function as repressors or enhancers akin to promoters (Figure 5B). The absence of the *PAL* allele “GCGCAC” in white *F. velutipes* prevented the activation of downstream lignin anabolism. Importantly, this study identified that the promoter of *FvbHLH1w* contained a “CATTTG-motif”, which is primarily a MYC response element. However, “CATTTG” is not a typical MYC element, which may be related to other transcription factors or regulatory factors. This distinction may elucidate the specific transcriptional activity of *FvbHLH1y* in the yellow cap and its role in the targeted accumulation of phenolic acid.

### 4.4. Overexpression of FvbHLH1 Can Regulate Phenylpropane Biosynthesis and Metabolism in Tobacco

The overexpression of *FvbHLH1* has been confirmed to activate key structural genes in the phenylpropanoid biosynthesis pathway in tobacco, as evidenced by protein–protein interaction software, which predicts an interaction between *FvbHLH1* and *PAL* (Figure 5D). Previously, research has demonstrated that the bHLH and MYB transcription factors can interact synergistically with structural genes such as *PAL*, *F3H,* and *DFR* to regulate anthocyanin biosynthesis. The activation of *FvbHLH1* was associated with the accumulation of primary metabolites, including saccharides and fatty acid compounds in tobacco. Conversely, there was a notable down-regulation of the levels of specific amino acids, such as l-proliyl-L-phenylalanine and l-aspartyl-L-phenylalanine. Collectively, these data have provided strong evidence to support the hypothesis that *FvbHLH1* acts as a regulatory role in the phenylalanine metabolic pathway, leading to the significant utilization of phenylalanine as a precursor molecule.

The quantitative analysis of the total phenolic acid content indicated that *FvbHLH1* induced an accumulation of phenolic acids in five transgenic tobacco lines, while concurrently elevating the content of ferulic acid (Figure 5E,F). This evidence further substantiates the involvement of *FvbHLH1* in the biosynthesis of phenolic acids. Although only primary mass spectrometry analyses were performed in this study, extensive targeted metabolomic investigations conducted in our previous work on plant anthocyanins suggested that the majority of relevant compounds were present in tobacco leaves. *FvbHLH1* is likely to engage in an interaction with MYB transcription factors in tobacco, thereby activating the flavonoid biosynthesis pathways and the accumulation of flavonoids. A significant decline in the phenylalanine levels confirms the activation of downstream pathways by *FvbHLH1*, and this is corroborated by both the primary metabolomics and MS data. Interestingly, small compound peaks corresponding to increased levels of chlorogenic acid (a class of phenolic acids) in transgenic tobacco were present (Figure S7), which provides additional credence to the hypothesis that *FvbHLH1* participates in the biosynthesis of downstream phenylalanine derivatives.

## 5. Conclusions

In this study, both metabolomic and transcriptomic analyses were employed to illustrate the phenotype of the cap in *F. velutipes*. Notably, the key compounds were identified, including α-linolenic acid, γ-linolenic acid, 2,5-dihydroxybenzaldehyde, protocatechuic acid ethyl ester, and ferulic acid. The insertion of the sequence “GCGCAC” in the *PAL* gene in the yellow variety leads to the activation of the lignin biosynthesis pathway, accompanied by extensive alterations in the phenolic acid production profiles. The distinction in the promoter induces the functional differentiation of *FvbHLH1* in *F. velutipes*. Specifically, *FvbHLH1* stimulates phenylalanine biosynthesis and metabolism in tobacco, facilitating the accumulation of flavonols and organic acids, while concurrently depleting significant levels of phenylalanine as a precursor.

## Data Availability

The transcriptomic data has been successfully uploaded to NCBI. PRJNA646543: RNA-seq data of *Flammulina velutipes*. SAMN15538725: *Flammulina velutipes_w1* (TaxID: 38945); SAMN15538726: *Flammulina velutipes_w2* (TaxID: 38945); SAMN15538727: *Flammulina velutipes_w3* (TaxID: 38945); SAMN15538728: *Flammulina velutipes_y1* (TaxID: 38945); SAMN15538729: *Flammulina velutipes_y2* (TaxID: 38945); SAMN15538730: *Flammulina velutipes_y3* (TaxID: 38945). All data generated or analyzed during this study are included within the article and its Supplementary Materials.

## Supplementary Materials

The following supporting information can be downloaded at: https://www.mdpi.com/article/10.3390/jof9111063/s1: Figure S1: KEGG annotation classification of all genes. Figure S2: GO annotation classification of all genes. Figure S3: KEGG annotation classification of DEGs. Figure S4: The KEGG enrichment histogram of conjoint analysis of DEGs and accumulated metabolites. Figure S5: Alignment of the open reading frame sequence of *PAL* gene in white (*PALw*) and yellow (*PALy*) *F. velutipes* alleles. Figure S6: The nucleotide sequence alignment of *PAL* gene promoter sequence in white (*PALwP*) and yellow (*PALyP*) *F. velutipes*. Figure S7: MS total ion flow diagrams of wild-type and overexpressed *FvbHLH1* tobacco crude extracts. Figure S8: Phylogenetic tree analysis of *FvbHLH1*, and the gene structure maps of *FvPAL* and *FvbHLH1*. Table S1: Oligo nucleotide primers used in this work. Table S2: The different accumulation of chemical compounds in white and yellow samples. Table S3: Summary of transcriptome sequencing data in the white and yellow. Table S4: Distribution of the lengths of the assembled unigenes. Table S5: The annotation of assembled unigenes. Table S6: The differently expression unigenes in white and yellow samples. Table S7: The relative transcript level of the unigenes relative to phenylalanine, tyrosine, and tryptophan biosynthesis. Table S8: All MYB and bHLH transcription factors in *F. velutipes*.
