# Supplementary material for: FvbHLH1 Regulates the Accumulation of Phenolic Compounds in the Yellow Cap of Flammulina velutipes
Source: J Fungi (Basel). 2023 Oct 30;9(11):1063. doi: 10.3390/jof9111063 (PMC10672597; doi:10.3390/jof9111063)
Supplement: Supplementary file 1 [file jof-09-01063-s001.zip › Table S1.pdf]

Table S1. Oligo nucleotide primers used in this work

Gene	Forward primer(5'-3')	Reverse primer(5'-3')	Function
FvbHLH1-1	ATGGTCATGTCGCTCCCCAAC	CTACATCATGGCAAAATTGGA	Cloning *FvbHLH1* cDNAs and gDNA
FvbHLH1-2	GGggatccATGGTCATGTCGCTC CCCAACCCCAG	GGtcgagaCTACATCATGGCAAA ATTGGCAATATCC	Construction of 35S-*FvbHLH1*
FvbHLH1-3	CAATCACCGCTCTCGTCCGCG	CTACATCATGGCAAAATTGGCA	Cloning *FvbHLH1* promoter
NtActin	AATGATCGGAATGGAAGCTG	TGGTACCACCACTGAGGACA	
NtPAL	ATGCTAAAACTGTAA	CTTGGTTCTCCTATG	
NtC4H	TGAGTTTGATTTTGG	GATTTCTCCCTTCTG	
Nt4CL	TTTCTTTCTTGGAGT	ATGACGGTTCTTACT	
NtMYC2	TCCGTCTTCTTGTGC	CGGTGTTCTTGCTCA	Amplifying Tobacco Actin gene transc ripts (as internal control of qRT-PCR)
NtF5H	AGATGAGAAAAGTGTGTG	TAGCAAGAGTGGTGAATA	
NtCoMT	TCTCAACTCAGAACCCAG	GCAACAGAAACACCATCA	
NtCalB	ATGGCGACGCTTGAATACAC	TCGCCGATGGTGGGTCCAAT	
