# Supplementary material for: FvbHLH1 Regulates the Accumulation of Phenolic Compounds in the Yellow Cap of Flammulina velutipes
Source: J Fungi (Basel). 2023 Oct 30;9(11):1063. doi: 10.3390/jof9111063 (PMC10672597; doi:10.3390/jof9111063)
Supplement: Supplementary file 1 [file jof-09-01063-s001.zip › Table S3.pdf]

Table S3. Summary of transcriptome sequencing data in the white and yellow

Sample	Total Raw Reads(M)	Total Clean Reads(M)	Total Clean Bases(Gb)	Clean Reads Q20(%)	Clean Reads Q30(%)	Clean Reads Ratio(%)
white 1	38.12	38.12	5.72	98.72	95.81	100
white 2	41.48	41.48	6.22	98.62	95.61	100
white 3	40.79	40.79	6.12	98.53	95.54	99.99
yellow 1	39.86	39.86	5.98	98.52	95.45	99.99
yellow 2	41.83	41.83	6.27	98.64	95.8	99.99
yellow 3	40.23	40.23	6.03	98.62	95.56	100
