# Supplementary material for: FvbHLH1 Regulates the Accumulation of Phenolic Compounds in the Yellow Cap of Flammulina velutipes
Source: J Fungi (Basel). 2023 Oct 30;9(11):1063. doi: 10.3390/jof9111063 (PMC10672597; doi:10.3390/jof9111063)
Supplement: Supplementary file 1 [file jof-09-01063-s001.zip › Table S4.pdf]

Table S4. Distribution of the lengths of the assembled unigenes

Sample	Total Number	Total Length	Mean Length	N50	N70	N90	GC(%)
white 1	27296	42563326	1559	2228	1604	883	51.24
white 2	27982	44539778	1591	2275	1632	899	51.22
white 3	29968	45318413	1512	2244	1599	838	51.17
yellow 1	36471	53377683	1463	2106	1532	824	51.12
yellow 2	35697	52695747	1476	2104	1535	840	51.15
yellow 3	34438	51749840	1502	2112	1543	863	51.18
