# Supplementary material for: FvbHLH1 Regulates the Accumulation of Phenolic Compounds in the Yellow Cap of Flammulina velutipes
Source: J Fungi (Basel). 2023 Oct 30;9(11):1063. doi: 10.3390/jof9111063 (PMC10672597; doi:10.3390/jof9111063)
Supplement: Supplementary file 1 [file jof-09-01063-s001.zip › Table S5.pdf]

Table S5. The annotation of assembled unigenes

Values	Nr	Nt	Swissprot	KEGG	KOG	Pfam	GO	Intersection	Overall
Number	162,413	36,975	106,171	116,156	101,874	139,184	57,053	13,824	166,873
Percentage	84.97%	19.34%	55.55%	60.77%	53.30%	72.82%	29.85%	7.23%	87.30%
