# Supplementary material for: FvbHLH1 Regulates the Accumulation of Phenolic Compounds in the Yellow Cap of Flammulina velutipes
Source: J Fungi (Basel). 2023 Oct 30;9(11):1063. doi: 10.3390/jof9111063 (PMC10672597; doi:10.3390/jof9111063)
Supplement: Supplementary file 1 [file jof-09-01063-s001.zip › Table S7.pdf]

Table S7. The relative transcript level of the unigenes relative to Phenylalanine, tyrosine and tryptophan biosynthesis

Structural gene	GeneID	Length	white-expression	yellow-expression	log2FoldChange(yellow/white)	Up/Down-Regulation	Sum of Log2
PAL	CL3691.Contig2_All	3003	0.442323	2993.154	12.72428	Up	15
	CL3691.Contig4_All	2979	622.1693	3022.567	2.280395	Up	
C4H	Unigene27196_All	411	0.334439	1.332035	1.993816	*	1.99
C3H	Unigene12973_All	1697	39.34255	26.2812	0.244378	*	1.36
	Unigene12976_All	1891	0.289143	34.65341	6.905072	Up	
	CL3508.Contig3_All	2332	6181.362	5134.318	-0.26775	*	
	CL3508.Contig2_All	2183	281.1041	562.2962	1.000226	Up	
	Unigene12972_All	1715	5.837032	4.074761	0.713639	*	
CoMT	Unigene12971_All	1721	1.013321	0.273802	0.403418	*	13.52
	Unigene2916_All	3744	46.3737	27.97909	-0.72896	*	
	Unigene2910_All	3183	616.6343	988.5564	0.680908	*	
	CL1458.Contig1_All	1723	82.77354	130.1623	0.65307	*	
	Unigene2909_All	5797	189.735	253.6515	0.418862	*	
	Unigene2907_All	3184	9.499301	1.289609	-2.88089	*	
	Unigene2907_All	3184	9.499301	1.289609	-2.88089	*	
	Unigene17080_All	3693	200.3534	32.04092	-2.64456	Down	
	Unigene2905_All	5719	797.6435	169.8751	-2.23127	Down	
	Unigene2911_All	3121	1153.687	528.5393	-1.12617	Down	
	Unigene2913_All	3169	0.379436	201.1935	9.050511	Up	
	Unigene2912_All	3127	0.402114	1033.06	11.32703	Up	
	Unigene2908_All	3070	43.42445	644.2152	3.890963	Up	
calB	CL13674.Contig4_All	1706	0.998528	914.8328	9.83949	Up	10.07
	CL13674.Contig3_All	1959	267.119	327.5751	0.294343	*	

	CL13674.Contig2_All	1875	836.5163	513.3183	-0.70454	*	
	CL13674.Contig1_All	1950	330.8781	518.4085	0.64779	*	
	CL8683.Contig6_All	1709	264.6909	65.22786	-2.02075	Down	
	CL8683.Contig1_All	1810	551.7176	1136.496	1.04259	Up	
F5H	CL15078.Contig3_All	2020	1182.398	829.9538	-0.51061	*	15.45
	CL15078.Contig2_All	1947	1.633497	1051.264	9.329945	Up	
	CL15078.Contig1_All	1995	0.314531	61.69547	7.615821	Up	
	Unigene18009_All	4108	1.61458	689.6841	8.738633	Up	
	CL9008.Contig6_All	5345	2300.055	371.6414	-2.62969	Down	
	CL9008.Contig5_All	5342	125.9634	53.86931	-1.22547	Down	
4CL	Unigene36077_All	2210	1.892833	22.59112	3.577137	Up	14.63
	CL5715.Contig1_All	2006	566.7678	277.6832	-1.02932	Down	
	CL13311.Contig2_All	2024	0.296097	61.86351	7.706876	Up	
	CL5715.Contig2_All	1922	36.82474	26.06918	-0.49833	*	
