# Supplementary figures and images for: FvbHLH1 Regulates the Accumulation of Phenolic Compounds in the Yellow Cap of Flammulina velutipes
Source: J Fungi (Basel). 2023 Oct 30;9(11):1063. doi: 10.3390/jof9111063 (PMC10672597; doi:10.3390/jof9111063)

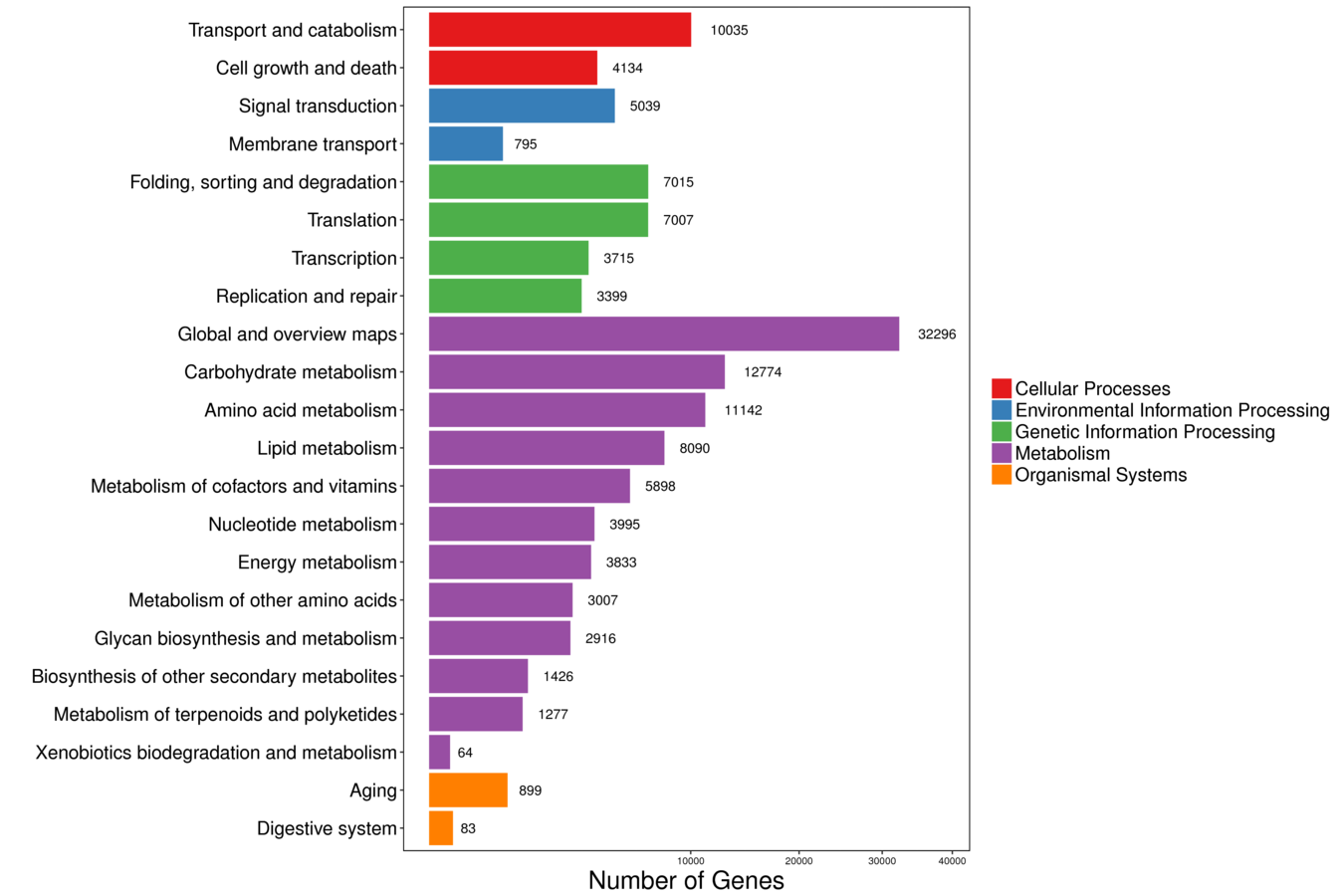

Supplement: Supplementary file 1 [file jof-09-01063-s001.zip › Figure S1.tif]

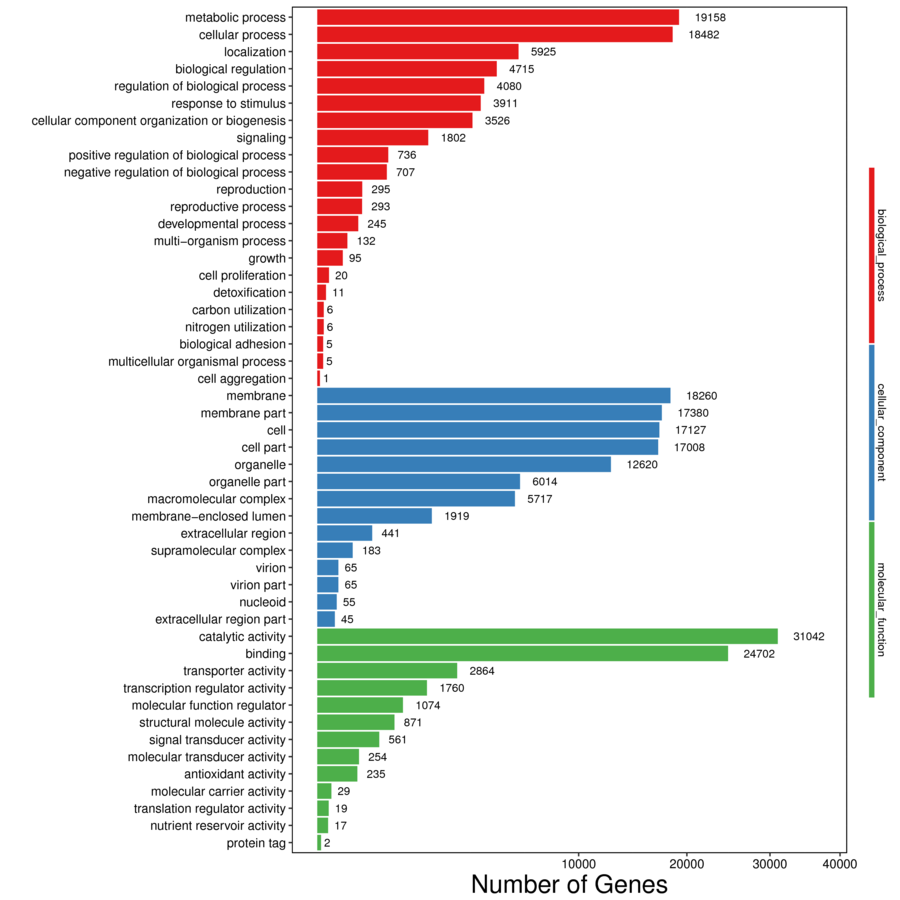

Supplement: Supplementary file 1 [file jof-09-01063-s001.zip › Figure S2.tif]

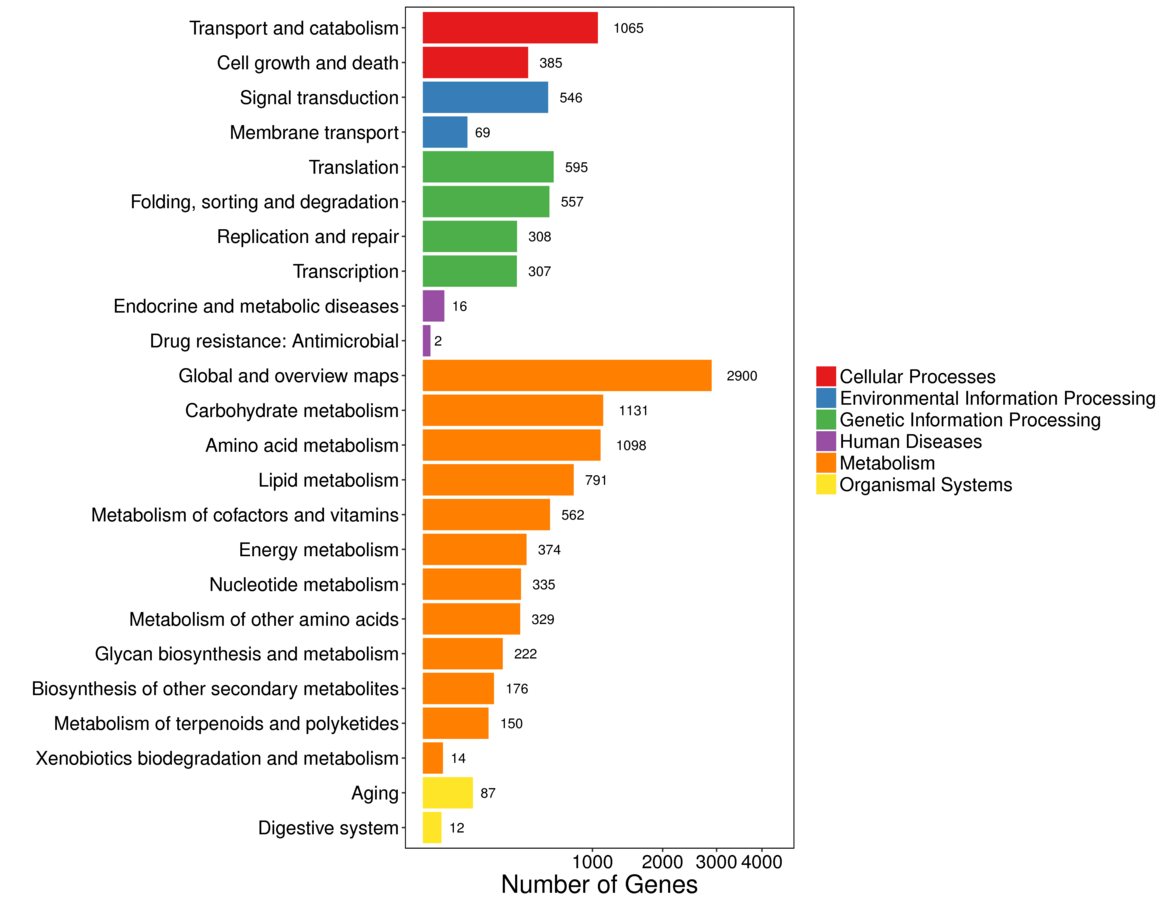

Supplement: Supplementary file 1 [file jof-09-01063-s001.zip › Figure S3.tif]

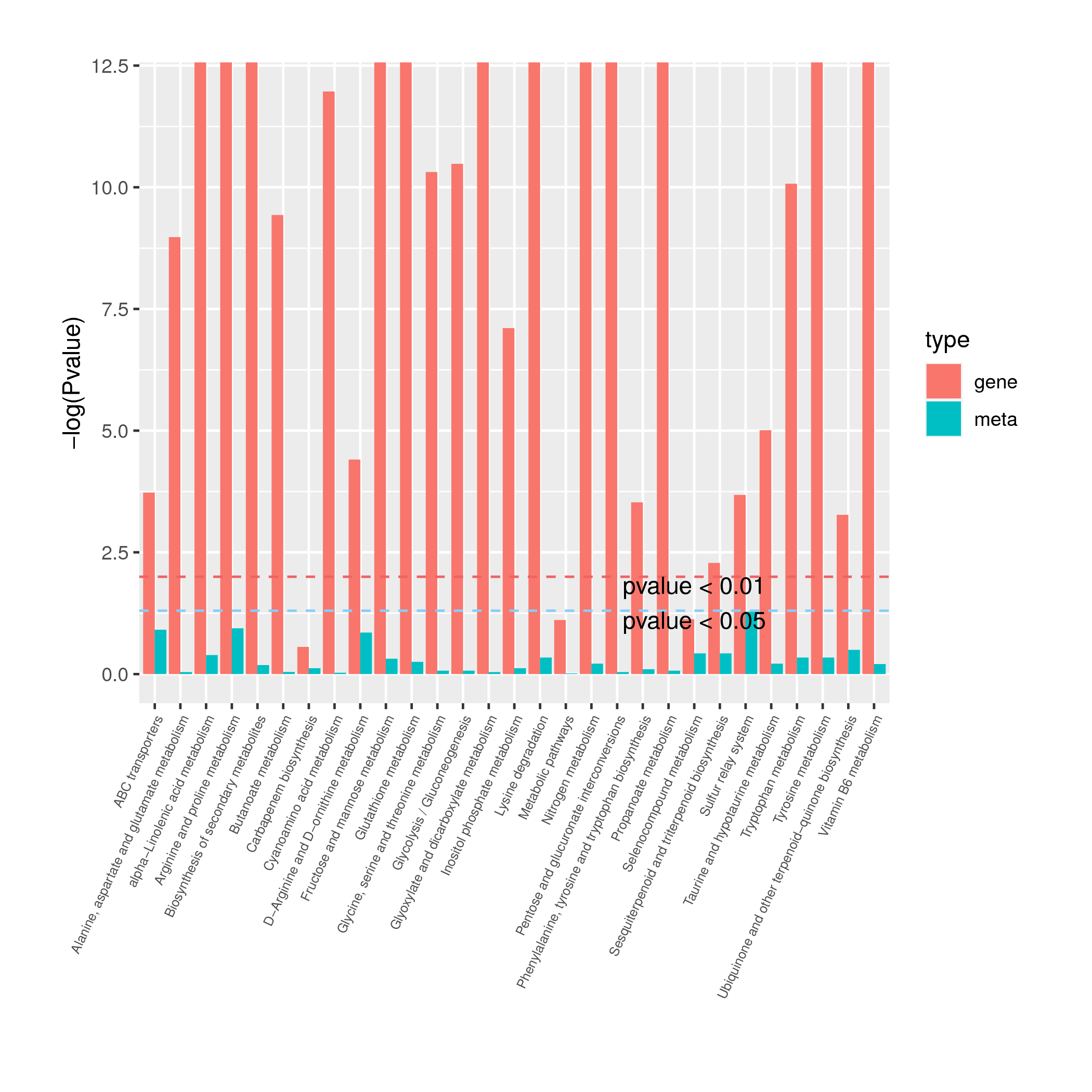

Supplement: Supplementary file 1 [file jof-09-01063-s001.zip › Figure S4.tif]

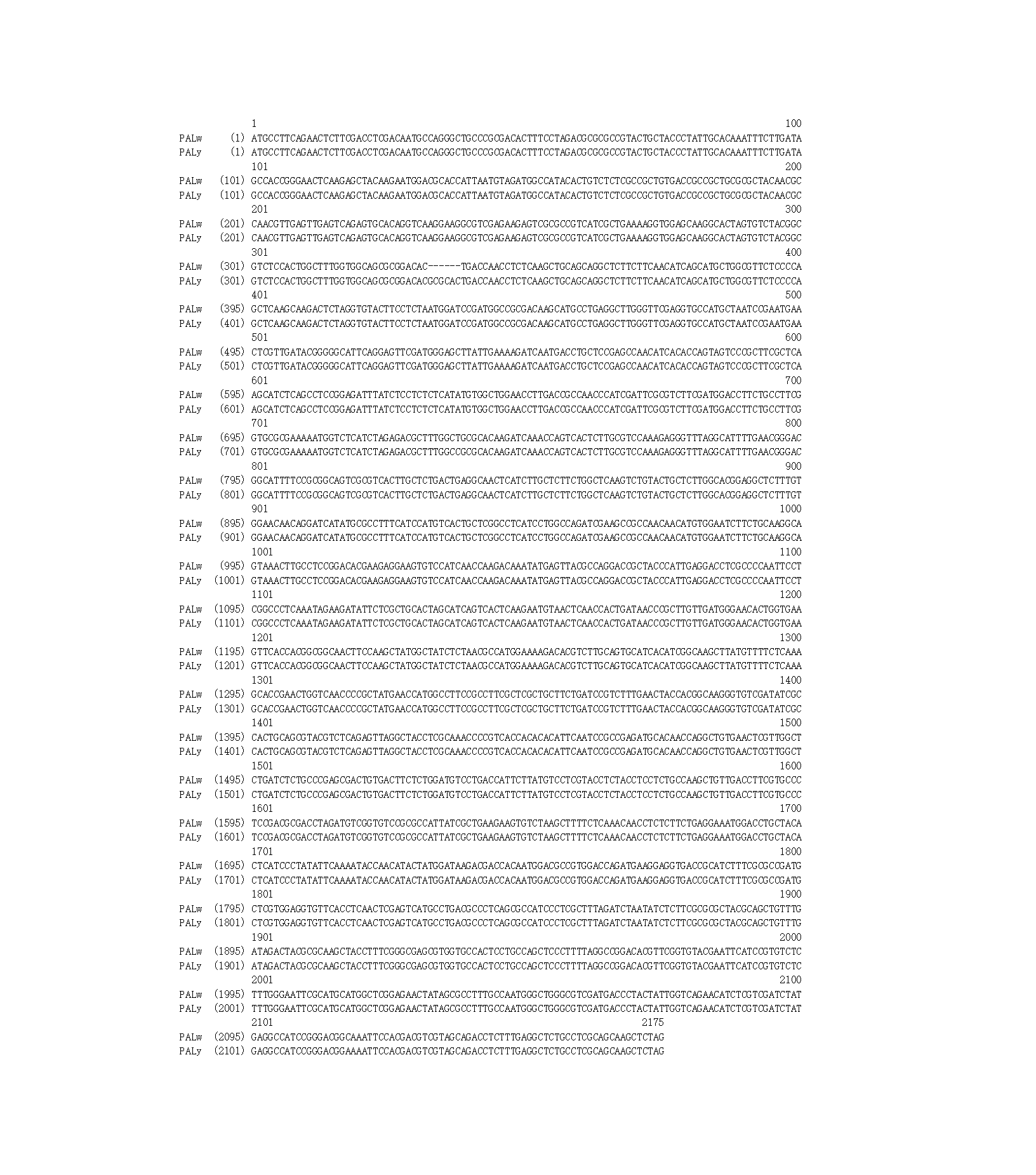

Supplement: Supplementary file 1 [file jof-09-01063-s001.zip › Figure S5.tif]

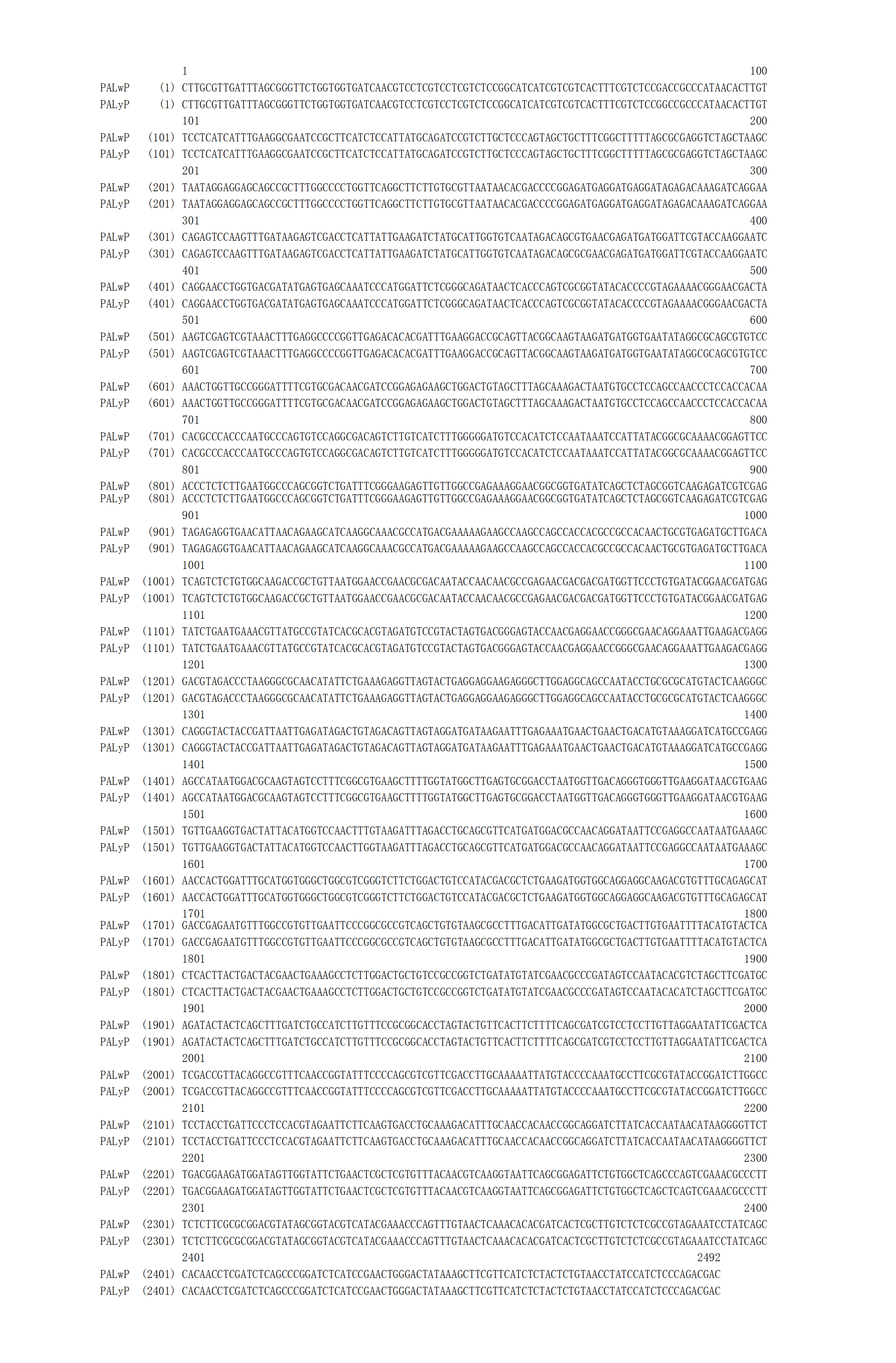

Supplement: Supplementary file 1 [file jof-09-01063-s001.zip › Figure S6.tif]

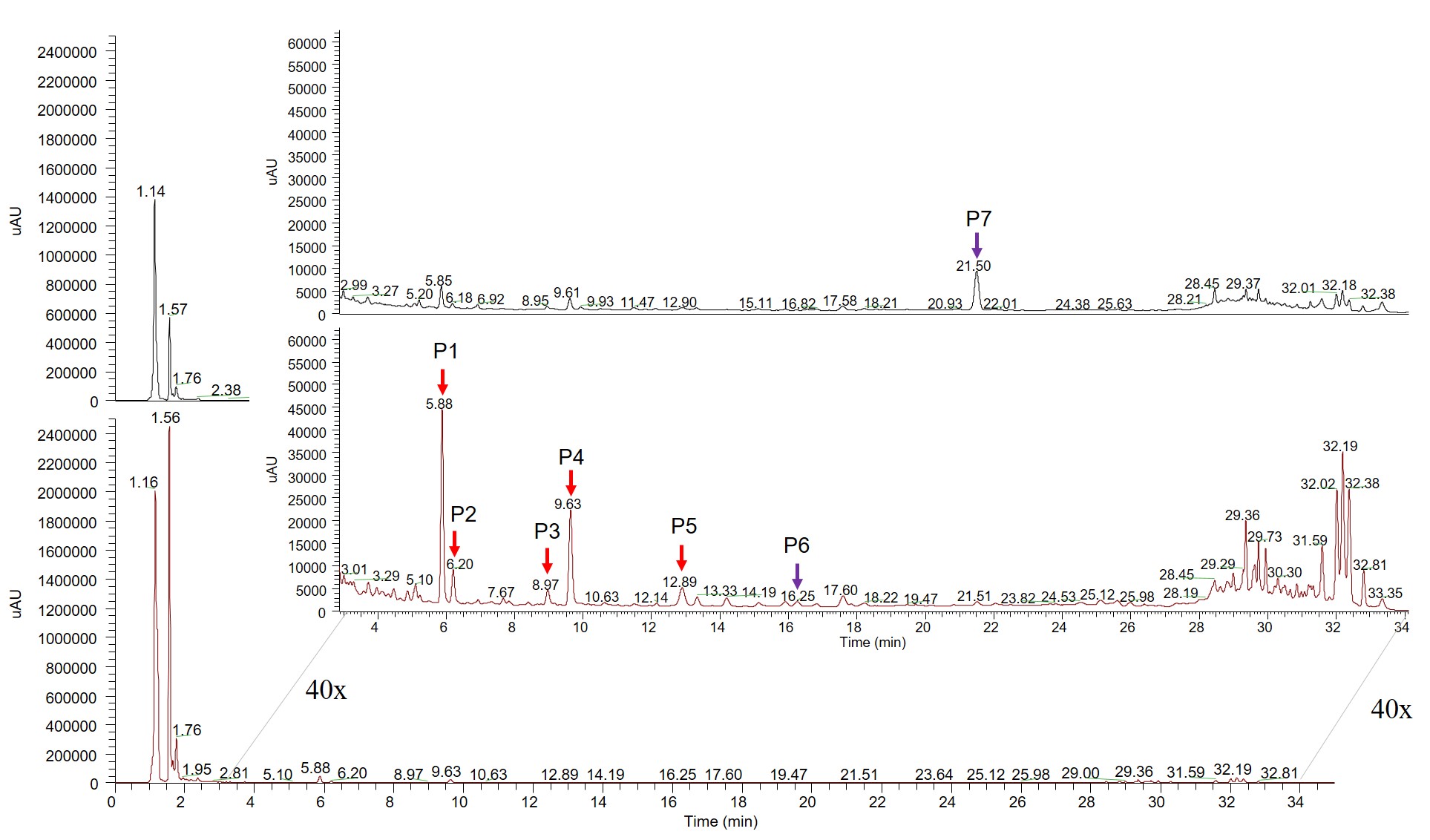

Supplement: Supplementary file 1 [file jof-09-01063-s001.zip › Figure S7.tif]

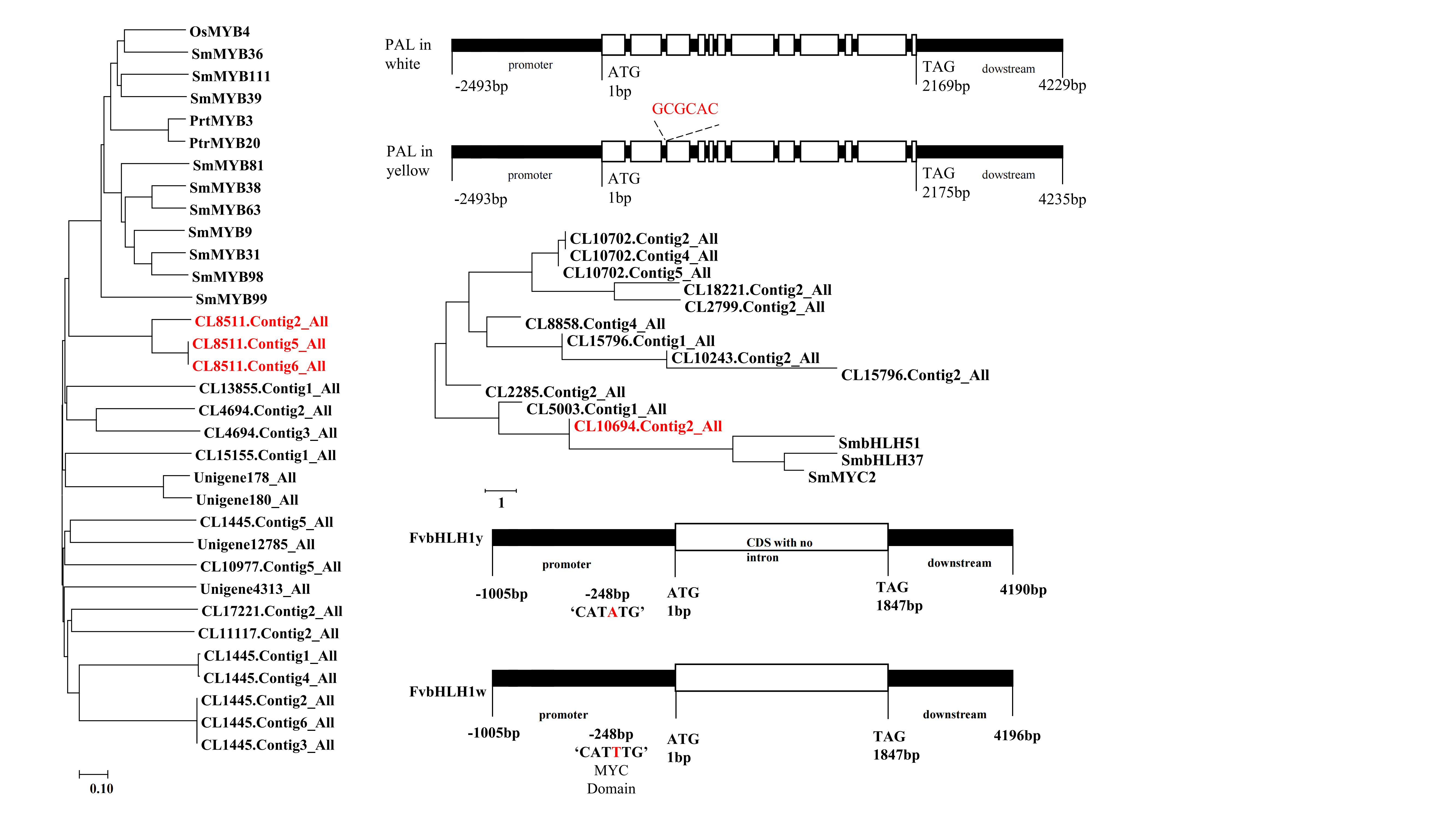

Supplement: Supplementary file 1 [file jof-09-01063-s001.zip › Figure S8.tif]
